# A Two-Way Road: Antagonistic Interaction Between Dual-Species Biofilms Formed by *Candida albicans/Candida parapsilosis* and *Trichophyton rubrum*

**DOI:** 10.3389/fmicb.2020.01980

**Published:** 2020-09-04

**Authors:** Letícia Morais Garcia, Caroline Barcelos Costa-Orlandi, Níura Madalena Bila, Carolina Orlando Vaso, Larissa Naiara Carvalho Gonçalves, Ana Marisa Fusco-Almeida, Maria José Soares Mendes-Giannini

**Affiliations:** ^1^Clinical Mycology Laboratory, Department of Clinical Analysis, School of Pharmaceutical Sciences, São Paulo State University (UNESP), Araraquara, Brazil; ^2^School of Veterinary, Eduardo Mondlane University, Maputo, Mozambique

**Keywords:** polymicrobial biofilms, *Candida albicans*, *C. parapsilosis*, dermatophytes, antagonistic interaction, dermatomycosis, *Trichophyton rubrum*

## Abstract

Dermatomycoses include superficial fungal infections of the skin and its appendages. *Trichophyton rubrum*, *Candida albicans*, and *Candida parapsilosis* are some of the most prevalent species that cause dermatomycoses. Several studies show a variable predominance of *Candida* spp. in relation to dermatophytes, especially in onychomycosis and the possibility of isolating both from the same site. The ability of dermatophytes to form biofilms recently been explored and there is currently no evidence on the involvement of these filamentous fungi in multi-species biofilms. Thus, this study aims to investigate the probable dual-species interaction between *T. rubrum* and *C. albicans* and *T. rubrum* and *C. parapsilosis* biofilms, considering variable formation conditions, as well as the susceptibility of these dual-species biofilms against terbinafine and efinaconazole. Three conditions of formation of dual-species biofilms were tested: (a) the suspensions of *T. rubrum* and *Candida albicans* or *C. parapsilosis* placed together; (b) suspensions of *C. albicans* and *C. parapsilosis* added the pre-adhesion of *T. rubrum* biofilms; (c) after the maturation of *T. rubrum* sessile cells. In the first and second conditions, the quantification of metabolic activities, biomass, and polysaccharide materials of mixed biofilms tended to resemble *Candida* monospecies biofilms. In the third condition, the profiles were modified after the addition of *Candida*, suggesting that *T. rubrum* biofilms served as substrate for the development of *Candida* biofilms. Scanning electron microscopy showed *Candida* predominance, however, numerous blastoconidia were noted, most evident in the conditions under which *Candida* was added after the pre-adhesion and maturation of *T. rubrum* biofilms. Despite the predominance of *Candida*, the presence of *T. rubrum* appears to inhibit *C. albicans* filamentation and *C. parapsilosis* development, confirming an antagonistic interaction. Fungal burden assays performed when the biofilms were formed together confirmed *Candida* predominance, as well as susceptibility to antifungals. Further studies will be needed to identify the components of the *Candida* and *T. rubrum* biofilm supernatants responsible for inhibiting dermatophyte growth and *C. albicans* filamentation.

## Introduction

Fungal infections of the skin, nails (onychomycosis), and cutaneous-mucous surfaces, known as dermatomycosis, are caused by several etiological agents and are an important cause of morbidity (Rotta et al., 2012). Among dermatomycosis, dermatophytosis, caused by dermatophytes, are the most prevalent worldwide, affecting 20 to 25% of the population (Havlickova et al., 2008; Zhan and Liu, 2017). *Trichophyton rubrum* is the microorganism most frequently associated with superficial mycoses (Costa-Orlandi et al., 2012, 2014; Zhan and Liu, 2017; Celestrino et al., 2019). Previous studies have reported that some strains of *T. rubrum* have a high dissemination power in relation to other dermatophytes, showing the dominance of this microorganism among dermatophytosis (Ghannoum et al., 2013).

Regarding yeasts of the genus *Candida*, *C. albicans* is the pathogen most commonly isolated from superficial mycoses. However, the incidence of infections by non-*C. albicans* species, such as *C. parapsilosis*, *C. tropicalis, C. krusei*, *C. glabrata*, and *C. guilliermondii*, has been increasing considerably (Sardi et al., 2013). Among, yeasts of *C. parapsilosis* are considered emerging pathogens and are commensal microorganisms of human skin. This microorganism is able to form biofilms in catheters and is transmitted through physical touch to neonates and in the hemodialysis systems used by chronic renal patients, thereafter evolving into bloodstream infections (Trofa et al., 2008; Pires et al., 2011, 2012).

Clinical practice cases and unpublished studies have demonstrated that it is possible to isolate one or more microorganisms from several infection sites in superficial mycoses, with a marked predominance of dermatophytes in comparison to yeasts of the genus *Candida* (Ghannoum et al., 2000; Aquino et al., 2007; Costa-Orlandi et al., 2012). Other studies indicate a higher prevalence of yeasts, especially *C. parapsilosis* in relation to *C. albicans* and *T. rubrum* in patients with onychomycosis (Pontes et al., 2002; Chadeganipour et al., 2010; Costa-Orlandi et al., 2012).

One of the major virulence factors of *Candida* is its ability to form biofilms (Ramage et al., 2012), which has also been reported recently in dermatophytes (Costa-Orlandi et al., 2014). Biofilms consist of communities of cells embedded in a polymeric extracellular matrix. These communities can be comprised of microorganisms of different species, genera or even kingdoms with a highly complex organization, allowing them to perform various functions, including nutrition, excretion, growth, communication, and protection, with greater efficiency (Ramage et al., 2009, 2012; Costa-Orlandi et al., 2017). Unlike planktonic cells, those found in biofilms have different morphologies depending on their location in the community (Ramage et al., 2012). For example, the morphology of *C. albicans* cells in biofilms vary from that of yeasts, hyphae, pseudohyphae, and germ tube, while *C. parapsilosis* consists mainly of yeasts and few pseudohyphae (Chandra and Mukherjee, 2015). Communities formed by dermatophytes are made up of a dense network of interconnected hyphae covered and embedded by an extracellular matrix that can be dense or thin (Costa-Orlandi et al., 2014). Changes in morphology are often regulated by molecules used for communication between cells, through a mechanism known as *quorum-sensing*, which has been widely studied in *Candida* biofilms but not yet investigated in dermatophytes. In addition to morphology, quorum-sensing is responsible for biofilm development, cell population control, nutrient competition control, dissemination, and the establishment of new communities during infection (Wongsuk et al., 2016).

Compared to the information available on biofilms formed by yeasts of the genus *Candida*, information on biofilms formed by dermatophytes is greatly lacking. Studies have previously found dermatophytes cohabiting with yeasts of the genus *Candida* and other microorganisms in superficial infections (unpublished data). This has also been widely observed in clinical practice. Therefore, the formation of mixed biofilms between these fungi is likely since biofilms provide many advantages for the microorganisms involved and rarely involve only one species (Costa-Orlandi et al., 2017).

There has been an increase in the incidence of mixed infections, namely among dermatophytes with yeasts or even non-dermatophyte filamentous species (Gawaz and Weisel, 2018). This increase has been accompanied by reports of antifungal resistance and, consequently, an increase in therapeutic failures (Gupta and Foley, 2019). Kravvas et al. (2018) found that the majority of routine dermatological infections were in the form of biofilms. Although this has yet to be proven in the case of dermatophytes, biofilm formation has been attributed to the occurrence of onychomycosis, which is often refractory to antifungal treatment and has a high rate of recurrence (Gupta et al., 2016; Gupta and Foley, 2019). The heterogeneity of species within biofilms makes it difficult to characterize the contribution of each microorganism to the pathogenesis and maintenance of infection and disease (dos Santos et al., 2016; Iñigo and Del Pozo, 2018).

Finally, in multi-species biofilms, it is also essential to consider the intercellular communications that occur via quorum-sensing molecules, which allow one species to identify and react to the presence of another. Therefore, these mediators are also able to coordinate the collective behavior of microorganisms in these communities (Demuyser et al., 2014).

There is currently a lack of research demonstrating the involvement of dermatophytes in multi-species biofilms. Thus, this work aims to investigate the probable dual-species interaction between the biofilms of *T. rubrum* and *C. albicans* and *T. rubrum* and *C. parapsilosis*. We will take into account variable conditions of formation to evaluate the susceptibility of these dual-species biofilms against two potent last generation antifungals, both of which have a proven efficacy against these microorganisms alone.

## Materials and Methods

### Microorganisms

In this work, strains of *C. albicans* SC 5314, *C. parapsilosis* ATCC 22019, and *T. rubrum* ATCC MYA-4438 were used. All strains belonged to the collection of the Clinical Mycology Laboratory of the Department of Clinical Analysis, of the School of Pharmaceutical Sciences at São Paulo State University (UNESP). Strains of *C. albicans* and *C. parapsilosis* were maintained on Sabouraud Dextrose Agar (SDA) (Kasvi) (40 g/L), comprised of a mixture of peptic digestion of animal tissue (10 g/L) and pancreatic digestion of casein (10 g/l) and agar (15 g/L), at 35°C for 48 h. The dermatophytes were then kept on malt extract agar (Kasvi), comprised of 2% peptone from animal tissue (Sigma-Aldrich), 2% glucose (Synth) and 2% agar (Kasvi) at pH 5.7, incubated at 28°C for 7 days or until sporulation (Costa-Orlandi et al., 2014; Jacob et al., 2015; Vila et al., 2015).

### *In vitro* Formation of Monospecies Biofilms

The *Candida* biofilms were formed according to Pierce et al. (2008), with some modifications. Colonies of the stock cultures of *C. albicans* SC5314 and *C. parapsilosis* ATCC 22019 strains were subcultured in Sabouraud Dextrose broth (Kasvi) and incubated at 150 rpm and 35°C for 24 h. A fungal suspension was prepared in RPMI 1640 medium supplemented with L-glutamine without sodium bicarbonate (Gibco) and buffered with MOPS (Sigma-Aldrich) with 2% glucose (Synth) at a final concentration of 1 × 10^6^ cells/mL. Two hundred microliters and one thousand microliters were dispensed in the wells of the 96- and 24-well plates (Kasvi), respectively. The plates were incubated at 37°C for 24 h and 48 h (maturation period) without shaking. A period of 72 h was also added to observe the behavior of biofilms for later standardization in dual-species biofilms, as the maturation period of *T. rubrum* biofilms corresponds to 72 h (Costa-Orlandi et al., 2014).

*T. rubrum* biofilms were formed as described by Costa-Orlandi et al. (2014). Briefly, *T. rubrum* ATCC MYA-4438 was subcultured in Malt Extract agar and incubated at 28°C for 7 days. The colonies were covered with 5 ml sterile of 0.85% saline, and conidia were gently detached with the aid of a sterile swab. The supernatant was placed in 15-mL conical tubes (Corning) and left to stand for 15–20 min to allow the hyphae to settle, such that only microconidia remained in the supernatant. The microconidia were counted in a hemocytometer and the suspension was adjusted using sterile saline (0.85%) to obtain a final concentration of 1 × 10^6^ cells/mL. Subsequently, 200 μL and 1000 μL of the adjusted inoculum were dispensed in the 96- and 24-well plates, respectively. The plates were incubated at 37°C for 4 h without shaking for pre-adhesion of the biofilms. The supernatant was then removed and 200 μL and 1000 μL of RPMI 1640 medium was added to the wells of the 96- and 24 well plates, respectively. The plates were re-incubated at 37°C for up to 72 h. These biofilms were also formed at 96 h and 120 h for subsequent comparison with one of the conditions proposed for dual-species biofilms.

### *In vitro* Formation of Dual-Species Biofilms

The fungal suspensions of all microorganisms were prepared at a final concentration of 1 × 10^6^ cells/mL when plated. Mixed biofilms were formed under the following conditions: (a) the *T. rubrum*/*C. albicans* and *T. rubrum*/*C. parapsilosis* suspensions were dispensed in 96- and 24-well plates at the same time in a proportion of 1:1. The plates were then incubated at 37°C for up to 72 h; (b) *Candida* suspensions were added after the initial adhesion phase of *T. rubrum* (4 h), followed by incubation at 37°C for up to 72 h; (c) *Candida* suspensions were added after the maturation period of *T. rubrum* biofilms (72 h) before incubating the plates at 37°C for an additional 24 h and 48 h. The culture medium was renewed every 24 h.

### Determination of the Metabolic Activity of Biofilms Using XTT Reduction Assay (2,3-Bis (2-Methoxy-4-Nitro-5-Sulfophenyl)-5-[Carbonyl (Phenylamino)]-2H-Tetrazolium Hydroxide)

Biofilms were formed as described above at different time intervals (24 h to 120 h). A solution of XTT (Thermo Fisher Scientific) was prepared at a final concentration of 1 mg/mL in phosphate buffered saline (PBS) and menadione (1 mM) (Sigma-Aldrich). After removing the supernatant and washing the biofilms with PBS, 50 μL of XTT solution + 4 μL of menadione solution were added. The plates were subsequently incubated at 37°C for 3 h and measured using a Biotek Epoch^TM^ 2 spectrophotometer at a wavelength of 490 nm (Martinez and Casadevall, 2007; Costa-Orlandi et al., 2014).

### Quantification of Biomass by Crystal Violet (CV) Assay

The biomass quantification test was performed according to Marcos-Zambrano et al. (2014), with some modifications. Briefly, biofilms were formed in 96-well plates (Kasvi) at different incubation times. The resulting supernatants were removed and the remaining biofilms were washed with PBS. Two hundred microliters of methanol (Vetec) were added to each well for 15 min. The methanol was discarded and the plates were dried at room temperature for 45 min. After drying, 200 μL of 0.1% violet crystal solution (Dinamica) was dispensed into the wells for 20 min. The resulting supernatant was carefully aspirated and the wells were washed with distilled water until the complete removal of excess dye. Finally, 200 μL of 33% acetic acid (Synth) was added and plates were read using a spectrophotometer (Biotek Epoch^TM^ 2 Microplate Spectrophotometer) at a wavelength of 570 nm. The resulting absorbance values were directly proportional to the amount of biomass.

### Quantification of the Extracellular Matrix and Other Polysaccharides by Safranin Staining

The polysaccharide material and extracellular matrices of mono- and multi-species biofilms were stained with 50 μL of 1% safranin solution for 5 min after removing the supernatant and washing the wells with PBS. The plates were washed with 200 μL of sterile 0.85% saline solution until the supernatant became clear. Lastly, the absorbance of the plates was measured using a Epoch^TM^ 2 microplate spectrophotometer at 492 nm (Seidler et al., 2008; Costa-Orlandi et al., 2014).

### Scanning Electron Microscopy (SEM)

Mono- and dual-species biofilms were formed in 24-well plates and incubated until the maximum time determined in each condition (as described above). The resulting supernatants were removed and the remaining biofilms were fixed with 800 μL of 2.5% glutaraldehyde solution (Sigma-Aldrich) for 1 to 2 h at 4°C. The glutaraldehyde solution was removed from the wells and the biofilms were dehydrated using increasing concentrations of alcohol (50–100%). After drying, the bottom of the plates was cut using a scalpel. The resulting samples were mounted on aluminum cylinders containing carbon strips and evaporated under high vacuum (Denton Vacuum Desk V; Jeol Ltd., Moorestown, NJ, United States) for gold coating. The topographical characteristics of the biofilms were analyzed by electron microscopy (JSM-6610 Series Scanning Electron Microscope; Jeol Ltd., United States) at the School of Dentistry of UNESP at Araraquara, SP, Brazil (Martinez et al., 2010; Costa-Orlandi et al., 2014; Li et al., 2019).

### Colony Forming Units Counting Assay (CFU/mL) in Selective Media

For the *C. albicans*/*T. rubrum* and *C. parapsilosis*/*T. rubrum* biofilms (1:1), the colony forming units were counted in selective media. Monospecies biofilms were used as a control. After the formation of biofilms in 96-well plates, the culture media were discarded for the removal of poorly adherent cells. Two hundred microliters of sterile PBS were added, and the biofilms were detached from the wells with the aid of sterile tips. The contents of the wells were transferred to 1.5 ml microtubes (Kasvi) and vortexed until the complete dispersion of the biofilm cells. Serial dilutions were performed in PBS and 10 μL aliquots of the diluted content were plated in 60 × 15 mm Petri dishes containing sterile glass beads and two selective media: CHROMagar^TM^ *Candida* (Difco; BD Biosciences) for the isolation of yeasts and Mycosel agar (Difco; BD Biosciences) for the isolation of *T. rubrum*. The plates were incubated at 28 and 37°C until the formation of colony forming units (dos Santos et al., 2016).

### Activity of Terbinafine and Efinaconazole Against Mono- and Dual-Species Biofilms

The activity of terbinafine (Sigma-Aldrich) and efinaconazole (Valeant Pharmaceuticals) was verified against mono- and dual-species biofilms formed using the previously described conditions. Biofilms were formed in 96-well plates. After 72 h, the supernatants were removed and 200 μL of the different concentrations of the terbinafine (0.03 to 32 mg/L) and efinaconazole (0.5 to 256 mg/L) working solutions were prepared in RPMI-1640 medium supplemented with 2% glucose. Two controls were performed: a control for the sterility of the medium (negative control) and a control for growth without treatment (positive control). The plates were incubated without shaking at 37°C for an additional 48 h. Cell viability was evaluated using XTT reduction assay (Pierce et al., 2008). A reduction of at least 50% of metabolic activity was considered an inhibition compared to the metabolic activity of biofilms without treatment.

### Statistical Analysis

At least three independent experiments were performed in triplicate for all tests, except for the colony-forming unit count assay. In this test, the assays were conducted with two independent experiments in triplicate. Data were subjected to statistical analysis using analysis of variance (ANOVA) with Bonferroni’s *post hoc* test in GraphPad Prism 5.0 software. *P* < 0.05 was considered statistically significant.

## Results

### Determination of the Metabolic Activity of Biofilms by XTT Reduction Assay

#### Monospecies Biofilms

**FIGURE 1:**
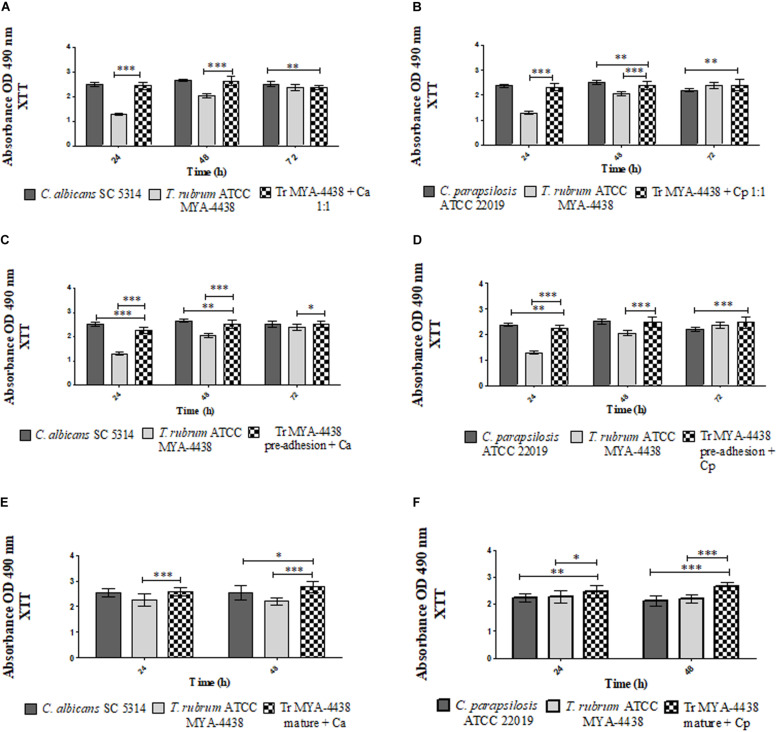
Comparison between the metabolic activities of monospecies biofilms of *T. rubrum* ATCC MYA 4438, *C. albicans* SC 5314, and *C. parapsilosis* ATCC 22019 and the dual-species biofilms formed by *C. albicans*/*T. rubrum* and *C. parapsilosis*/*T. rubrum* under three different conditions: (1) the suspensions of *Candida* and *T. rubrum* were placed together at a proportion of 1:1 **(A,B)**; (2) *Candida* suspensions added after the pre-adhesion of *T. rubrum* biofilm **(C,D)**; (3) *Candida* suspensions added after the maturation of the *T. rubrum* biofilm, followed by an additional incubation of 24 h and 48 h **(E,F)**. Statistical significance: *p < 0.05, **p < 0.01; ***p < 0.0001. Tr, *T. rubrum*; Ca, *C. albicans*; Cp, *C. parapsilosis.*

The metabolic activities of the *Candida* monospecies biofilms rose significantly until 48 h (*p* < 0.0001) (Figure 1). At 72 h, there was a significant decline in the metabolism of both species (*p* < 0.0001). In *T. rubrum* biofilms, there was a significant increase in the metabolic activities for up to 72 h (*p* < 0.0001). After this period, the metabolic activities remained constant until 96 h, followed by a slight decay in 120 h (*p* < 0.05). However, during the periods of decay, there was still a considerable amount of living cells in the biofilm, indicating the beginning of the inhibition of mature biofilm formation.

#### Dual-Species Biofilms Formed Together (1:1)

The metabolic activities of the dual-species biofilms of *T. rubrum*/*C. albicans* and *T. rubrum*/*C. parapsilosis* were compared to the monospecies biofilms of each strain (Figure 1). Figure 1A shows the dual-species biofilm formed between *T. rubrum* ATCC MYA-4438 and *C. albicans* SC 5314. Until 48 h, the dual-species biofilm showed a higher metabolism than the monospecies biofilm of *T. rubrum* (*p* < 0.0001), similar to *C. albicans* biofilm. At 72 h, the metabolic activities of the dual-species biofilms were similar to that of the monospecies biofilms of *T. rubrum* and lower compared to that of *C. albicans* (*p* < 0.01).

A similar behavior was observed between the dual-species biofilms formed by *T. rubrum* ATCC MYA-4438 and *C. parapsilosis* ATCC 22019 (Figure 1B). At 24 h, the metabolic activity of the dual-species biofilm was equal to that of *C. parapsilosis* and higher than *T. rubrum* (*p* < 0.0001). At 48 h, the dual-species biofilm showed higher metabolic activities than the *T. rubrum* biofilm (*p* < 0.0001) and lower than *C. parapsilosis* (*p* < 0.01). At 72 h, the dual-species biofilms showed a superior metabolic activity compared to *C. parapsilosis* (*p* < 0.01) and similar levels of metabolic activity as *T. rubrum*.

#### Dual-Species Biofilms Formed After *T. rubrum* Pre-adhesion

The formation of dual-species biofilms in which *Candida* suspensions were added after the pre-adhesion of *T. rubrum* are shown in Figures 1C,D. Figure 1C shows the dual-species biofilm formed between *T. rubrum* ATCC MYA-4438 and *C. albicans SC 5314*. After 24 h, the mixed biofilm showed a higher metabolic activity than the *T. rubrum* biofilm (*p* < 0.0001) and lower than that of *C. albicans* (*p* < 0.0001). After 48 h of incubation, the dual-species biofilm showed lower metabolic activity than the *C. albicans* biofilm (*p* < 0.01) and higher than the *T. rubrum* biofilm (*p* < 0.0001). In 72 h, the dual-species biofilm exhibited a higher metabolic activity than the *T. rubrum* monospecies biofilm (*p* < 0.05), but similar to *C. albicans*.

When the *C. parapsilosis* suspension was added after *T. rubrum* pre-adhesion (Figure 1D), the metabolic activity of the dual-species biofilm was lower to that of *C. parapsilosis* (*p* < 0.01) and superior to that of *T. rubrum* (*p* < 0.0001) until 24 h. At 48 h, the dual-species biofilm showed metabolic activity similar to that of the *C. parapsilosis* monospecies biofilm and superior to that of *T. rubrum* (*p* < 0.0001). At 72 h, the mixed biofilm showed a higher metabolic activity than the *C. parapsilosis* biofilm (*p* < 0.0001), but similar to *T. rubrum*.

#### Dual-Species Biofilms Formed After Maturation of *T. rubrum* Biofilm

For this condition, *T. rubrum* biofilms were incubated until 72 h, corresponding to the maturation time. During this period, *Candida* suspensions were also added. The plates were then incubated for an additional 24 h and 48 h. Therefore, the plates incubated for 24 h and 48 h shown in Figures 1E,F correspond to the same periods after the addition of *Candida* and 96 h and 120 h of *T. rubrum* biofilm incubation. In relation to the additional incubation time of the *T. rubrum* biofilms, it is worth noting that the metabolic activities remained constant until 96 h, with a slight decay at 120 h, which can result in an inhibition phase (data not shown). Figure 1E shows that 24 h after the addition of the *C. albicans* suspensions, there was a significant increase in the metabolic activities of the dual-species biofilms compared to those of *T. rubrum* monospecies biofilms (*p* < 0.0001). The same result was observed after 48 h compared to the *C. albicans* (*p* < 0.05) and *T. rubrum* (*p* < 0.0001) monospecies biofilms. When the *C. parapsilosis* suspension was added, the mixed biofilms showed higher values of metabolic activities than the monospecies biofilm of *C. parapsilosis* (*p* < 0.01) and *T. rubrum* (*p* < 0.05) after 24 h (Figure 1F). After 48 h, the metabolic activities of mixed biofilms were superior to both monospecies biofilms of *C. parapsilosis* and *T. rubrum* (*p* < 0.0001).

### Quantification of Biomass by Crystal Violet (CV) Staining

#### Monospecies Biofilms

**FIGURE 2:**
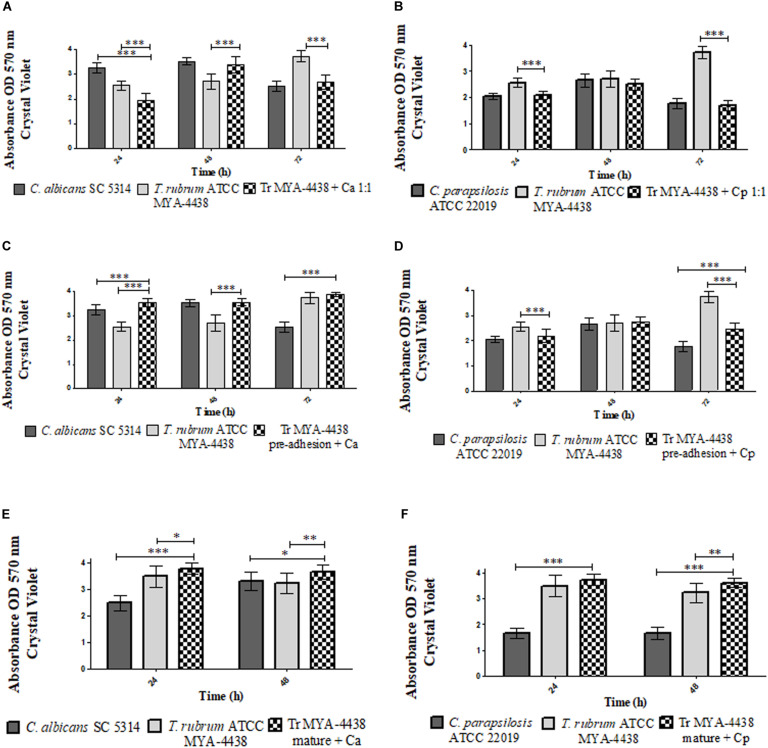
Comparison between the biomasses produced by the monospecies biofilms of *T. rubrum* ATCC MYA 4438, *C. albicans* SC 5314, and *C. parapsilosis* ATCC 22019 and by the dual-species biofilms formed by *C. albicans*/*T. rubrum* and *C. parapsilosis*/*T. rubrum* under three different conditions: (1) suspensions of *Candida* and *T. rubrum* together at a proportion of 1:1 **(A,B)**; (2) *Candida* suspensions added after the pre-adhesion of *T. rubrum* biofilm **(C,D)**; (3) *Candida* suspensions added after the maturation of the *T. rubrum* biofilm, followed by an additional incubation of 24 h and 48 h **(E,F)**. Statistical significance: *p < 0.05; **p < 0.01; ***p < 0.0001. Tr, *T. rubrum*; Ca, *C. albicans*; Cp, *C. parapsilosis.*

The monospecies biofilms formed by *C. albicans* and *C. parapsilosis* indicated an increase in the development of biomass within the first 48 h (*p* < 0.0001). After this period (at 72 h), there was a significant decrease (*p* < 0.0001) in the absorbance measurements. Regarding *T. rubrum* biofilms, an increase in the biomass was observed until 72 h (*p* < 0.0001). After this period, there was no statistical significance between the absorbances up to 96 h, corresponding to a stationary phase, followed by a significant decay in 120 h (*p* < 0.001) (Figure 2). These results are in agreement with the findings of the XTT reduction assay.

#### Dual-Species Biofilms Formed Together (1:1)

The mass of the dual-species *C. albicans*/*T. rubrum* and *C. parapsilosis*/*T. rubrum* biofilms formed after the addition of fungal suspensions are shown in Figures 2A,B, respectively. In the first interaction (Figure 2A), the dual-species biofilm at 24 h showed a lower biomass than the monospecies biofilms (*p* < 0.0001). At 48 h, the biomass had significantly increased (*p* < 0.001). Compared to the monospecies biofilms, its development was similar to that of *C. albicans* and superior to the *T. rubrum* biofilm (*p* < 0.0001). The biomass decayed after 72 h of incubation, similar to the *C. albicans* biofilm and in contrast to the *T. rubrum* biofilm, whose biomass increased (*p* < 0.0001).

In the dual-species biofilms formed between *C. parapsilosis* and *T. rubrum* (Figure 2B), the biomass was similar to that of the monospecies *C. parapsilosis* biofilm and inferior to the *T. rubrum* biofilms (*p* < 0.0001) at 24 h. After 48 h of incubation, the biomass absorbance measurements were similar to both types of monospecies biofilms. At 72 h, the biomass of the dual-species biofilm was similar to that of *C. parapsilosis* and lower than *T. rubrum* (*p* < 0.0001).

#### Dual-Species Biofilms Formed After *T. rubrum* Pre-adhesion

When *C. albicans* suspensions were added after *T. rubrum* pre-adhesion (Figure 2C), the dual-species biofilms showed higher biomasses compared to both types of monospecies biofilms (*p* < 0.0001) at 24 h of incubation. At 48 h, the biomass of the dual-species biofilm was similar to *C. albicans* and higher than *T. rubrum* (*p* < 0.0001). At 72 h of incubation, the biomass was similar to that of the *T. rubrum* biofilm, which was superior to that of *C. albicans* (*p* < 0.0001).

In the dual-species biofilm formed between *T. rubrum* and *C. parapsilosis* (Figure 2D), the biomasses of dual-species biofilms were similar to the *C. parapsilosis* biofilms and inferior to *T. rubrum* (*p* < 0.0001) at 24 h. At 48 h, the biomass of the dual-species biofilms was similar to that of both monospecies biofilms. However, after 72 h, the mass of the biofilm was higher than that of *C. parapsilosis* (*p* < 0.0001) and lower than *T. rubrum* (*p* < 0.0001). These results indicate that *Candida* is predominant in this condition, although less than in the previous condition.

#### Dual-Species Biofilms Formed After Maturation of *T. rubrum* Biofilm

The biomass of dual-species biofilms formed after the addition of *Candida* suspensions for 24 h and 48 h in mature *T. rubrum* biofilms are shown in Figures 2E,F. After the addition of *C. albicans* (Figure 2E), the biomasses at 24 h and 48 h was superior to that of the monospecies biofilms of *T. rubrum* and *C. albicans* (*p* < 0.05). After 24 h of incubation with added *C. parapsilosis*, the biomass was similar to that of the *T. rubrum* biofilms and superior to the *C. parapsilosis* biofilms (*p* < 0.0001). After 48 h of incubation, the mass of the mixed biofilms was higher than the *C. parapsilosis* (*p* < 0.0001) and *T. rubrum* (*p* < 0.01) monospecies biofilms.

### Quantification of Extracellular Matrix and Other Polysaccharides by Safranin Staining

#### Monospecies Biofilms

**FIGURE 3:**
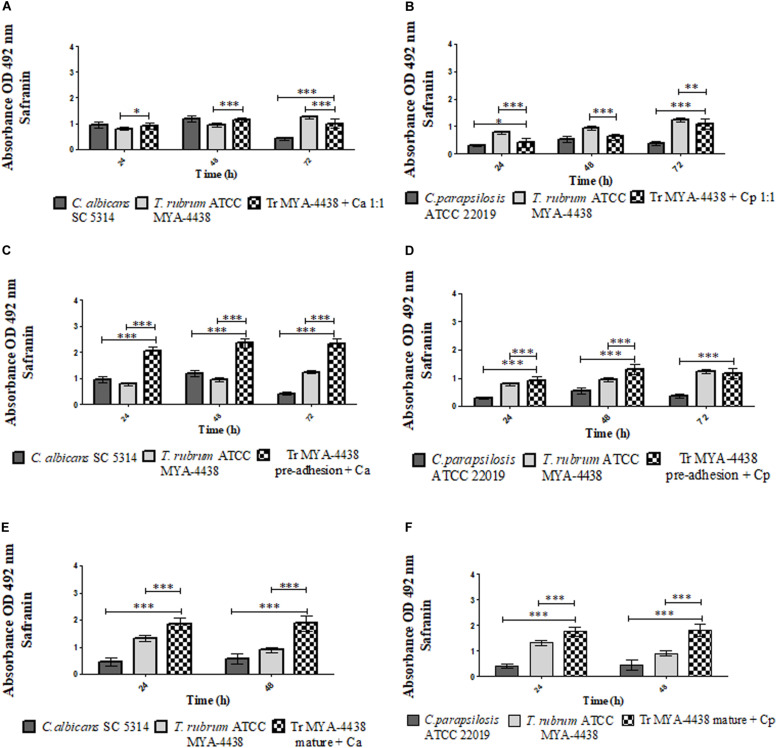
Comparison between the amounts of extracellular matrix and polysaccharide structures produced by the monospecies biofilms of *T. rubrum* ATCC MYA 4438, *C. albicans* SC 5314, and *C. parapsilosis* ATCC 22019 and by the dual-species biofilms formed by *C. albicans*/*T. rubrum* and *C. parapsilosis*/*T. rubrum* under three different conditions: (1) suspensions of *Candida* and *T. rubrum* placed together at a proportion of 1:1 **(A,B)**; (2) *Candida* suspensions added after the pre-adhesion of *T. rubrum* biofilm **(C,D)**; (3) *Candida* suspensions added after the maturation of the *T. rubrum* biofilm, followed by an additional incubation of 24 h and 48 h **(E,F)**. Statistical significance: *p < 0.05; **p < 0.01; ***p < 0.0001. Tr, *T. rubrum*; Ca, *C. albicans*; Cp, *C. parapsilosis.*

The monospecies biofilms were stained with safranin as a control in order to compare the amount of extracellular matrix and polysaccharide structures produced compared to the dual-species biofilms. In Figure 3, the production of polysaccharide material from *C. parapsilosis* ATCC 22019 and *C. albicans* SC 5314 increased until 48 h of incubation (*p* < 0.0001). After 72 h, decays were observed in both types of yeast biofilm (*p* < 0.0001). In *T. rubrum*, the production of polysaccharide material increased until 72 h (*p* < 0.0001) and remained stable until 96 h, before declining at 120 h (*p* < 0.0001). These results corroborate with the results obtained in both the XTT and crystal violet assays, suggesting a maturation period for *Candida* biofilms of 48 h and for *T. rubrum* biofilms of 72 h.

#### Dual-Species Biofilms Formed Together (1:1)

The dual-species biofilm resulting from the *T. rubrum* ATCC MYA-4438 and *C. albicans* SC 5314 suspensions (Figure 3A) showed similar amounts of polysaccharide structures as the *C. albicans* biofilm (*p* < 0.05) and superior to the dermatophyte monospecies biofilm (*p* < 0.0001) after 48 h. At 72 h, the amount of polysaccharide material was higher than that of the *C. albicans* biofilm and lower than the *T. rubrum* biofilm (*p* < 0.0001).

For the dual-species biofilm formed using *T. rubrum* ATCC MYA-4438 and *C. parapsilosis* ATCC 22019 (Figure 3B), the amount of polysaccharide material formed after 24 h of incubation was greater than that of the *C. parapsilosis* monospecies biofilms (*p* < 0.05) and lower than that of *T. rubrum* (*p* < 0.0001). After 48 h, the dual-species biofilm showed quantities of polysaccharide structures similar to those of the yeast biofilm and lower than the dermatophyte biofilm (*p* < 0.0001). The dual-species biofilm at 72 h showed extracellular matrix development superior to *C. parapsilosis* (*p* < 0.0001) and lower than *T. rubrum* (*p* < 0.01).

#### Dual-Species Biofilms Formed After *T. rubrum* Pre-adhesion

Figure 3C shows that the dual-species biofilm of *T. rubrum* ATCC MYA-4438 and *C. albicans* SC 5314 after *T. rubrum* pre-adhesion established a quantity of polysaccharide material superior to both monospecies biofilms (*p* < 0.0001) after 72 h of incubation.

When formed with *C. parapsilosis* (Figure 3D), the dual-species biofilm formed after pre-adhesion of the dermatophyte showed an extracellular matrix development superior to both monospecies biofilms (*p* < 0.0001). Lastly, after 72 h of incubation, the dual-species biofilm showed polysaccharide structures similar to that of the dermatophyte and superior to *C. parapsilosis* (*p* < 0.0001).

#### Dual-Species Biofilms Formed After Maturation of *T. rubrum* Biofilm

When the *C. albicans* suspension was added to the mature *T. rubrum* biofilm, the amounts of polysaccharide material of the dual-species biofilm were higher than those of monospecies biofilms of both *T. rubrum* and *C. albicans* (Figure 3E) (*p* < 0.0001) after an additional 24 h and 48 h of incubation. The same behavior was observed in the *C. parapsilosis* and *T. rubrum* dual-species biofilms (Figure 3F). These results support the use of dermatophytes as substrates for the development of *Candida* biofilms, and corroborate our quantification of the metabolic activities by XTT assays and of the biomass by crystal violet staining.

### Scanning Electron Microscopy (SEM)

**FIGURE 4:**
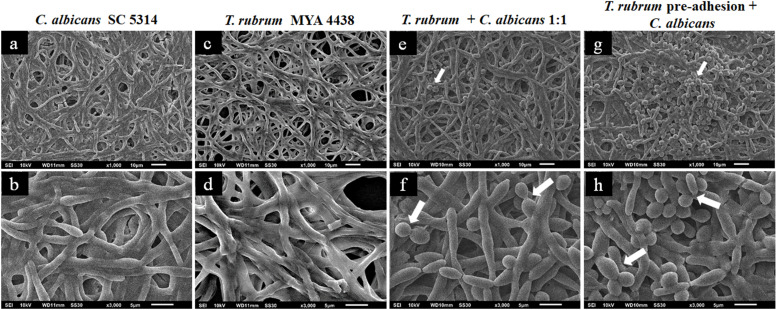
Scanning electron microscopies of *C. albicans* SC 5314 **(a,b)** and *T. rubrum* ATCC MYA 4438 **(c,d)** monospecies biofilms and dual-species biofilms of *C. albicans*/*T. rubrum* formed at a proportion of 1:1 **(e,f)** or formed with the addition of *C. albicans* suspensions after the pre-adhesion of *T. rubrum* biofilm **(g,h)**. White arrows denote the blastoconidia of *C. albicans*.

**FIGURE 5:**
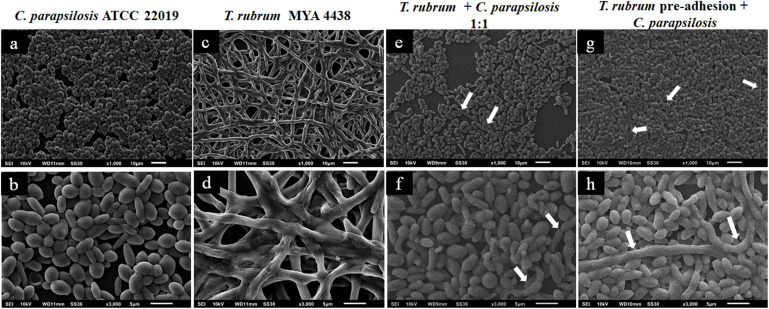
Scanning electron microscopies of monospecies biofilms of *C. parapsilosis* ATCC 22019 **(a,b)** and *T. rubrum* ATCC MYA 4438 **(c,d)** and dual-species biofilms of *C. parapsilosis*/*T. rubrum* formed at a proportion of 1:1 **(e,f)** or formed with the addition of *C. parapsilosis* suspensions after the pre-adhesion of *T. rubrum* biofilm **(g,h)**. White arrows denote the hyphae of *T. rubrum*.

**FIGURE 6:**
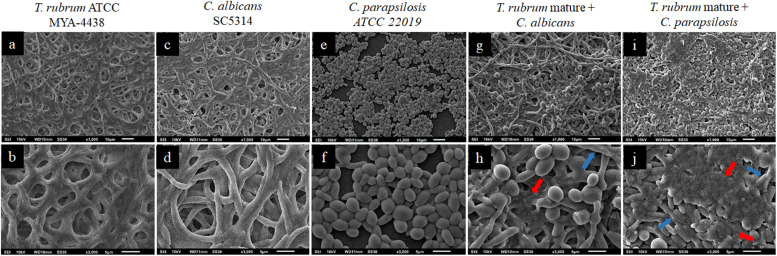
Scanning electron microscopies of *T. rubrum* ATCC MYA 4438 **(a,b)**, *C. albicans* SC 5314 **(c,d)**, and *C. parapsilosis* ATCC 22019 **(e,f)** monospecies biofilms and dual-species biofilms of *C. albicans*/*T. rubrum* **(g,h)** and *C. parapsilosis*/*T. rubrum* **(i,j)**, formed after the maturation of *T. rubrum* biofilm, followed by an additional 48 h incubation. It is possible to observe oval blastoconidia. Red arrows denote the extracellular matrices, while blue arrows denote hyphae.

The topographies of the dual-species biofilms of *C. albicans*/*T. rubrum* and *C. parapsilosis*/*T. rubrum* established under the three experimental conditions were analyzed by SEM with different magnifications (×1000 and ×3000) and compared to monospecies biofilms (controls) (Figures 4–6).

Figure 4 shows the topographies of the monospecies biofilms of *C. albicans* SC 5314 (Figures 4a,b) and *T. rubrum* ATCC MYA 4438 (Figures 4c,d), as well as their dual-species biofilms (1:1) (Figures 4e,f) and formation after *T. rubrum* pre-adhesion (Figures 4g,h). The biofilm of *C. albicans* SC 5314 (Figures 4a,b) was comprised of a dense hyphae network that developed from the yeasts, forming a complex three-dimensional structure. The same structures were observed in the *T. rubrum* ATCC-MYA 4438 biofilms, but appeared less dense than those in the *C. albicans* biofilms. Moreover, the polysaccharide material was at times dense and at other times thin, covering and connecting the hyphae network (Figures 4c,d). When the suspensions were placed together to form dual-species biofilms (Figures 4e,f), it was not possible to reliably distinguish between the hyphae formed by *C. albicans* and those formed by *T. rubrum* in the images. However, interestingly, a reasonable amount of blastoconidia (represented by the white arrows) was observed, suggesting that the interaction likely affected the mechanisms involved in the filamentation of *C. albicans*. The presence of blastoconidia indicates the probable predominance of *Candida* in relation to *T. rubrum*, more clearly seen in Figure 4f. When the *Candida* suspensions were added after 4 h of dermatophyte pre-adhesion (Figures 4g,h), similar results to those obtained in the previous condition were observed. However, numerous yeast blastoconidia were also observed, which reinforces our theory of the predominance of *Candida* in relation to *T. rubrum*. In addition, our findings indicate that the interaction with *T. rubrum* may somehow inhibit the filamentation of *C. albicans*. The greater prevalence of *C. albicans* compared to *T. rubrum* can be justified by the fact that yeasts have a faster metabolism than dermatophytes.

The topographies of the *C. parapsilosis* ATCC 22019 and *T. rubrum* MYA-4438 dual-species biofilms (1:1) after *T. rubrum* pre-adhesion are shown in Figure 5, along with their controls. Figures 5a,b show a predominance of blastoconidia and some rare pseudohyphae in the *C. parapsilosis* monospecies biofilm. The *T. rubrum* ATCC MYA 4438 biofilms are shown in Figures 5c,d. The interaction between *T. rubrum* and *C. parapsilosis* (Figures 5e,f) shows a predominance of yeast blastoconidia, but more dispersed than the control. We also observed poorly developed hyphae in the early stages, which suggests the inhibition of *C. albicans* filamentation and a low *T. rubrum* development, indicating an antagonistic interaction between the species studied.

A lower inhibition in the formation of mature biofilms, and, consequently, a more robust biomass, was observed when *C. parapsilosis* was added after *T. rubrum* pre-adhesion (Figures 5g,h). In addition, the *T. rubrum* hyphae were found to be more developed than in the previous condition (white arrows), although still in small quantities. Therefore, pre-adherence may benefit the development of the hyphae of dermatophytes, the latter of which serves as a substrate for the development of *C. parapsilosis* biofilms.

Figure 6 shows the topographies of the biofilms formed after the addition of the *C. albicans* and *C. parapsilosis* suspensions to the mature *T. rubrum* biofilms. The monospecies biofilms of *T. rubrum*, *C. albicans*, and *C. parapsilosis* are shown in Figures 6a–f, respectively. In the mixed biofilms of *C. albicans*/*T. rubrum* (Figures 6g,h), we observed numerous blastoconidia and a dense biomass, in addition to a greater amount of polysaccharide matrix (red arrows). The visualization of the mixed biofilms of *C. parapsilosis* and *T. rubrum* was better (Figures 6i,j). These findings reinforce our theory and corroborate our results for the quantification of biomass by CV and polysaccharide material by safranin staining.

### Colony Forming Units Counting Assay (CFU/mL) in Selective Media

**FIGURE 7:**
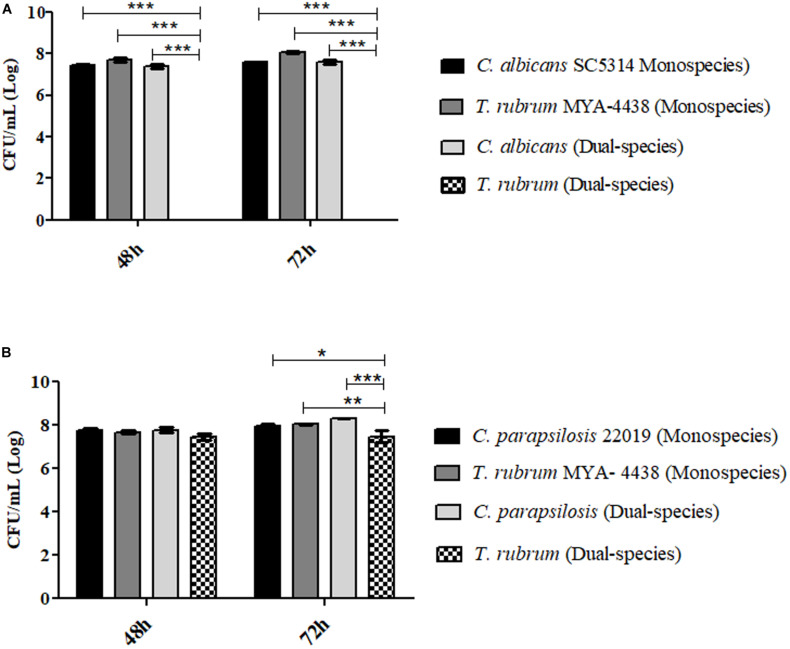
Log of colony forming units per milliliter [CFU/mL] (Log) isolated from mono- and dual-species *C. albicans* SC 5314 and *T. rubrum* ATCC MYA 4438 biofilms (1:1) **(A)** and isolated from mono and dual-species biofilms of *C. parapsilosis* ATCC 22019 and *T. rubrum* ATCC MYA 4438 **(B)** formed under the same conditions after 48 h and 72 h of incubation. Statistical significance: *p < 0.05, **p < 0.01, and ***p < 0.0001.

For the colony forming unit count (CFU) assay, only one condition for the formation of dual-species biofilms was chosen: *C. albicans* SC 5314/*C. parapsilosis* ATCC 22019 and *T. rubrum* ATCC MYA-4438 (1:1). Petri dishes containing CHROMagar^TM^ *Candida* (Difco; BD Biosciences) were used to isolate *C. albicans* from the mixed biofilm, while Mycosel agar (Difco; BD Biosciences) was used to isolate *T. rubrum* at different temperatures. Monospecies biofilms formed in the same period were used as controls. Figure 7A shows the isolation of *C. albicans* from the mixed biofilm with a fungal load after 48 h and 72 h of incubation, similar to that of the monospecies biofilm of the same yeast (no statistical significance). On the other hand, it was not possible to isolate *T. rubrum* from dual-species biofilms, confirming the inhibition of growth by yeast (*p* < 0.01) and reinforcing the results of the quantitative colorimetric tests, especially of CV, and SEM.

In contrast, *T. rubrum* was isolated from dual-species biofilms formed with *C. parapsilosis* (Figure 7B). The predominance of yeast in comparison with the dermatophyte was still evident, particularly after 72 h of incubation (*p* < 0.0001), confirming the antagonistic interaction. In terms of the controls, the fungal loads of *C. parapsilosis* and *T. rubrum* were found to be similar to those of dual-species biofilms after 48 h of incubation (no statistical significance). At 72 h, the fungal load of *T. rubrum* isolated from the dual-species biofilm was significantly lower than that of the monospecies biofilm (*p* < 0.01), whereas that of *C. parapsilosis* was similar. Therefore, we concluded that the interaction between *C. parapsilosis* and *T. rubrum* also culminated in the inhibition of *T. rubrum* growth.

### Activity of Terbinafine and Efinaconazole Against Mono- and Dual-Species Biofilms

To determine the minimum sessile inhibitory concentration (SMIC), a reduction of at least 50% of cell viability was considered in relation to positive controls. As shown in Table 1, both monospecies and dual-species biofilms were resistant to all tested concentrations of efinaconazole and terbinafine.

**TABLE 1:** Values of the minimum inhibitory concentration sessile (SMIC) expressed in mg/L for efinaconazole and terbinafine tested against the monospecies biofilms of *T. rubrum*, *C. albicans*, *C. parapsilosis*, and dual-species biofilms formed by *T. rubrum*/*C. albicans* and *T. rubrum*/*C. parapsilosis*.

Antifungals/fungi	*T. rubrum* MYA-4438	*C. albicans* SC 5314	*C. parapsilosis* ATCC 22019	*Tr*^∗^ MYA + *C. albicans*	*Tr*^∗^ MYA + *C. parapsilosis*
Efinaconazole	>256	>256	>256	>256	>256
Terbinafine	>32	>32	>32	>32	>32

**^∗^Tr: T. rubrum*.*

## Discussion

In mixed biofilms, interactions between different species can be synergistic, neutral, or antagonistic (dos Santos et al., 2016). Recent studies have shown that *Candida* yeast rarely exists in isolated form (Jack et al., 2015). Synergism is beneficial to all microorganisms involved, whereas antagonistic relationships can cause the death, damage, or inhibition of one microorganism by a product produced by the other. Finally, neutral relationships are indifferent for both species involved in the formation of the biofilm (Thein et al., 2009; Cabezón et al., 2016; Costa-Orlandi et al., 2017).

The development of multi-species biofilms can also be influenced by the sharing of nutrients available in the medium between different species. Therefore, the polymicrobial interactions in a biofilm are significantly modulated by these environmental elements (Thein et al., 2009; Cabezón et al., 2016). On the other hand, it is also essential to consider intercellular communications that occur through quorum-sensing molecules, which allow to coordinate the collective behavior of microorganisms in these communities (Demuyser et al., 2014).

Our results showed an *in vitro* antagonistic interaction between *C. albicans* and *C. parapsilosis* and the dermatophyte *T. rubrum* in RPMI medium. Although it is not known which of the three conditions tested actually occurred in the microbiome and in the pathogenesis of dermatomycoses, this antagonism may be caused by products secreted into the medium by both fungi, or even due to a lack of nutrients. To prevent the latter, the culture medium was changed every 24 h. The predominance of *Candida* was possibly due to its metabolism being more accelerated than that of *T. rubrum*, mainly in the first condition, in which the two microorganisms were added together. This condition can generate competition for adhesion sites. In the other conditions, the absorbance measurements corresponding to metabolic activities, biomasses, and polysaccharide materials were similar or sometimes superior to *T. rubrum* monospecies biofilms, which suggests that pre-adhesion or mature biofilms of *T. rubrum* may served as a substrate for the development of *Candida* biofilms. On the other hand, the *Candida* monospecies biofilms demonstrated differences in the quantity of the same parameters compared to dual-species biofilms. These assumptions were confirmed in the topographies of biofilms observed in SEM, which further evidenced the predominance of yeasts, the inhibition of the development of *T. rubrum* biofilms, and the inhibition of *C. albicans* filamentation, more evident in the conditions where there was a greater amount of the dermatophyte.

Regarding the quantitative tests of the monospecies biofilms, the results corroborated the literature, indicating that the ideal time for the maturation of the biofilms of *Candida* spp. (24 h or 48 h) depended on the species (Pierce et al., 2008). In comparison, the ideal maturation of *T. rubrum* is 72 h (Costa-Orlandi et al., 2014).

According to dos Santos et al. (2016), it is not possible to differentiate between the cellular metabolic activity of a single species in mixed biofilms based only on the XTT reduction assay. Therefore, it is necessary to make comparisons with monospecies biofilms. The same authors stated that the metabolic behavior of biofilms can vary depending on the microorganisms present in the microbial interaction. The presence of *C. krusei* in the mixed biofilm with *C. albicans* stimulated a reduction in cell viability compared to the viability observed in the monospecies biofilm formed by *C. albicans*, suggesting a possible inhibition of *C. albicans*.

Bandara et al. (2010b) used colorimetric assays with XTT to demonstrate that the presence of *Escherichia coli* in dual-species biofilms, after 48 h, decreased the cell viability of *C. parapsilosis*, without significant changes when compared to *C. albicans*.

The metabolic activities of biofilms were quantified through a colorimetric assay with a solution of XTT and menadione acting as an electron coupling agent. XTT is a yellow tetrazolium salt, which converts to orange formazan salts in the presence of metabolic activity. Many authors assume that this assay is an efficient way to indirectly quantify biofilms since the observed colorimetric change is proportional to the number of living cells (Costa-Orlandi et al., 2014; Marcos-Zambrano et al., 2014; Rosato et al., 2016). However, there are also studies indicating that, for *C. albicans* and *C. parapsilosis*, the results obtained with the XTT assay are not directly related to the number of live microorganisms (Kuhn et al., 2003). It is also important to consider that biofilms are surrounded by an extracellular matrix, which can impair the metabolic activity of the microorganisms if it limits the access of cells to nutrients and oxygen. Thus, other methods will be needed in addition to the XTT tests for the characterization of biofilms (Henriques et al., 2006; Costa-Orlandi et al., 2017).

Regarding the quantification of biomass by the crystal violet assay, previous studies have shown that the presence of *C. albicans* biofilms stimulated an increase in the biomass of heterotypic biofilms formed between *C. albicans*, *C. glabrata*, and *C. tropicalis*, indicating that the first species can provide a substrate for non-*C. albicans* species to adhere to acrylic materials (Pathak et al., 2012). The co-cultivation of *Streptococcus mutans* and *C. albicans* in dual-species biofilms resulted in better biomass growth for each species. This may have occurred due to metabolic interactions that provided additional nutrients for these microorganisms (Sztajer et al., 2014). In contrast, recent studies have shown an inhibitory effect on metabolic activity and biomass formation by *C. tropicalis*, *C. krusei*, and *C. parapsilosis* when co-incubated with probiotic bacteria *Lactobacillus gasseri* and *Lactobacillus rhamnosus* (Tan et al., 2018).

The crystal violet assay is based on the ability of this substance to penetrate the fungal cell wall and remain trapped in the cytoplasm. This quantification technique may have its reproducibility affected by the different growth conditions of the microbial biofilm and the different solvent concentrations used in the assay, among others. In addition, crystal violet is only able to quantify the biomass without distinguishing between dead and living cells, so this analysis must be complementary to other tests (Stepanovic et al., 2007; Pantanella et al., 2013).

In this study, the colony-forming unit counting test was used to complement the previous colorimetric assays, as well as the topographic observation by SEM, since these do not allow to specify the real constitution of the biofilm from a quantitative point of view. This assay confirmed the predominance of *C. albicans* and *C. parapsilosis* over *T. rubrum* in the only tested condition (1:1). Although laborious, many authors use this technique to isolate microorganisms within mixed biofilms and, unlike the XTT reduction assay, this technique is not influenced by the cellular metabolic state, directly enumerating the present cells (Jin et al., 2004; Jabra-Rizk et al., 2006; Bandara et al., 2010a; Fox et al., 2014; dos Santos et al., 2016).

Finally, regarding susceptibility tests, the findings in the present study corroborate the increased resistance that biofilms have against antimicrobial agents. In the dual-species interaction, although there is an antagonistic relationship between the biofilms of *Candida* and the dermatophyte, there is a prevalence of yeasts, making these dual-species biofilms resistant to all tested drug concentrations. The results obtained with the susceptibility tests of *T. rubrum* biofilms against terbinafine and efinaconazole corroborate the results obtained by our group and which are in preparation for publication. Previous studies have demonstrated the ineffectiveness of terbinafine against *C. albicans* biofilms, which agrees with the results presented in this work. However, the same authors demonstrated that terbinafine exerts activity against *C. parapsilosis* biofilms, contrary to what was found in the present study (Lam et al., 2016).

## Conclusion

This study provides unprecedented data related to the interaction between sessile communities formed by the three microorganisms that are most frequently isolated from dermatomycoses, one of the most prevalent mycoses worldwide. The findings show that *C. albicans*/*T rubrum* and *C. parapsilosis*/*T. rubrum* can grow and form biofilms together, albeit with a degree of antagonism on both sides. If, on the one hand, the growth of the dermatophyte is somewhat inhibited by the presence of yeasts, the presence of the filamentous fungi seems to proportionally inhibit one of the main virulence factors of *C. albicans*, namely filamentation, thereby decreasing the growth of *C. parapsilosis*. Even under condition in which the dermatophyte seems to serve as a substrate for the development of *Candida* biofilms, there is a certain antagonism for the same reasons as mentioned above. Therefore, this work opens several doors to the study of the interaction between *T. rubrum* biofilms and other microorganisms. Further studies are currently being conducted to identify potential substance(s) secreted in the supernatants of these mixed biofilms for use as broad-spectrum compounds. Additional studies should also be conducted using other species with different biofilm formation abilities in order to verify our findings.

## Data Availability Statement

All datasets generated for this study are included in the article/supplementary material.

## Author Contributions

CC-O, LMG, and NB drafted the manuscript. LMG, CC-O, NB, CV, and LNG performed the experiments. CC-O and MM-G designed and supervised the project. All authors participated in data analysis and critical revision of the manuscript and approved the final version.

## Conflict of Interest

The authors declare that the research was conducted in the absence of any commercial or financial relationships that could be construed as a potential conflict of interest. The handling editor declared a shared affiliation with the authors at the time of review.
